# Non-covalent interface engineering of multi-layer graphene cement composites using graphene oxide

**DOI:** 10.1016/j.isci.2026.115065

**Published:** 2026-02-17

**Authors:** Lei Fan, Chengtao Wu, Jinhao Zheng, Xiaohan Ji, Wang Zhang, Yunyun Tong, Qiannan Wang, Fangyuan Song, Hongwei Wang, Feng Li, Lucas Uzimaya, Guangyan Liu, Mengya Li

**Affiliations:** 1 School of Civil Engineering and Architecture, Zhejiang University of Science & Technology, Hangzhou, P.R. China; 2 Zhejiang International Science & Technology Cooperation Base for Low-carbon Building Material Technology with Recyclable Waste Resource, Zhejiang University of Science and Technology, Hangzhou 310023, China; 3 School of Civil Engineering and Architecture, Guilin University of Technology, Guilin, China; 4 CY Cergy Paris Université, L2MGC, EA 4114 Cergy 95000, France

**Keywords:** Applied sciences, Materials science, Nanomaterials

## Abstract

To address the critical demand for early strength in modern construction, this study proposes a novel MLGs/GO (multi-layer graphene/graphene oxide) nanocomposite system to synergistically enhance the early performance of cement mortar. Experimental results demonstrate that the optimized mixture (1.0% MLGs +0.025% GO) significantly improves mechanical properties, achieving a 7-day compressive strength of 46.91 MPa—an increase of over 58% compared to mortar with MLGs alone. The incorporation of GO effectively disperses MLGs, promotes the formation of key hydration products (CH, C-S-H, and AFt), and refines the microstructure. Molecular dynamics simulations reveal that GO introduces strong non-covalent interactions at the interface, replacing weak van der Waals forces and optimizing stress transfer between MLGs and the C-S-H matrix. These findings provide a clear multiscale mechanism for the synergistic enhancement and offer practical guidance for designing high-performance, early-strength cementitious materials.

## Highlights

• MLGs-Cement samples incorporating different cases of GO layer were prepared
• The addition of GO induces the non-covalent interaction within the MLGs/CSH composite
• GO and MLGs exert a synergistic effect that facilitates the hydration process of cement

## Graphical abstract

**Figure:**
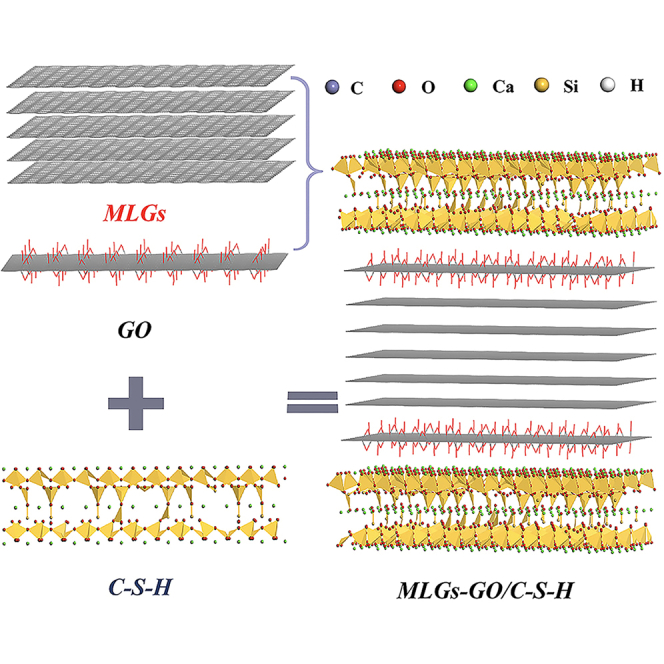

## Introduction

In recent years, with the continuous compression of engineering construction cycles and the rapid development of prefabricated buildings, the early mechanical properties of concrete materials have increasingly become an important index for evaluating their properties.^1^ Compared with the traditional idea of 28-day strength as the main design basis, current engineering practice pays more attention to the strength performance of concrete during the 3-day to 7-day curing cycle.^2^ This stage not only determines whether the structure can be demolded, loaded, or transferred to the next process in time, but is also directly related to construction efficiency and safety.^3^ From the perspective of material microevolution, cement hydration products, especially calcium hydrated silicate (C-S-H), act as the decisive factor in the final structural properties during the early nucleation and growth process.^4^^,^^5^^,^^6^ Studies have shown that promoting the rapid formation of C-S-H in the early stage not only helps to improve initial strength, but also optimizes microstructure, reduces porosity, and improves compactness.^7^^,^^8^ Therefore, it is of great significance to study the mechanism and means to improve the hydration degree and mechanical properties of cement in 3 and 7 days to ensure the quality of the project and promote the development of high-performance concrete materials.

In order to improve the early strength performance, many scholars have carried out research on cement clinker composition optimization, water-cement ratio control, admixture mixing, and mineral admixture activation, but traditional methods have limited room for improvement and are easily limited by environmental conditions.^9^^,^^10^ In recent years, nanomaterials have become a research hotspot for improving the early properties of cementitious materials due to their extremely high specific surface area, excellent dispersibility, and nucleation-promoting effect.^11^^,^^12^

The significant enhancement effect stems from a multi-scale synergistic mechanism triggered by unique physicochemical properties. Nanomaterials, possessing an extremely high specific surface area, provide abundant reactive interfaces for the hydration reaction of cement-based materials, which can greatly accelerate the initial hydration kinetics of cement minerals.^13^ Nanoparticles act as effective heterogeneous nucleation sites, significantly lowering the nucleation energy barrier for hydration products, thereby inducing the formation and deposition of a greater quantity and finer microstructure at early ages.^14^ Concurrently, particles at the nanoscale can fill the micron and sub-micron pores between cement particles, effectively improving the initial compactness and uniformity of the paste.^15^ Furthermore, the surfaces of nanomaterials can adsorb and transport key ions, locally altering the chemical equilibrium and ion migration rates in the pore solution, thus promoting the progress of early hydration reactions.^16^ The synergy among physical filling, chemical promotion, and crystallization regulation constitutes the core mechanism through which nanomaterials overcome the limitations of traditional technologies to efficiently improve the early-age performance of cement-based materials.

In the field of early-age enhancement of cement-based materials, mainstream materials such as nano-SiO_2_, nano-CaCO_3_, and carbon nanotubes (CNTs) each possess distinct mechanisms of action along with corresponding limitations. Nano-SiO_2_ primarily accelerates hydration by providing nucleation sites, while also contributing through micro-aggregate filling and pozzolanic effects, significantly improving early-age strength. However, its contribution to enhancing long-term toughness remains limited.^17^ Nano-CaCO_3_ mainly relies on physical filling and nucleation effects to improve later-age strength, yet it may even inhibit early strength development while also facing challenges such as uneven dispersion and dosage optimization.^18^ CNTs, owing to their high aspect ratio and excellent mechanical properties, show outstanding potential in bridging cracks and transferring stress. Nevertheless, their strong tendency to agglomerate in the highly alkaline environment of cement, coupled with poor dispersion stability, severely constrains the effective utilization of their reinforcing capabilities.^19^

Among them, multilayer graphene (MLG) and graphene oxide (GO) are extensively employed in the early reinforcement research of cementitious materials due to their good mechanical enhancement effect and interfacial interaction with hydration products. Silva et al.^20^ observed that the compressive strength of cementitious materials doped with 0.021% MLGs increased by 63.6% and 94.1% at 3 and 7 days, respectively. Through SEM observation, it was found that MLGs can fill the pores in the cementitious materials, and change the crack propagation path, ultimately boosting the material’s comprehensive performance. Liu et al.^21^ found that adding 0.04% GO to cement-based materials could increase the compressive strength by 46.9% at 6 h and the flexural strength by 121.4% at 100 min.

Although MLGs and GO have brought significant performance improvement potential to cement-based composite materials, their actual reinforcement effect is often limited by a common challenge, which is the agglomeration phenomenon of nanoparticles in the matrix. Due to the extremely high specific surface area and surface energy of the material, it is easy to aggregate into larger clusters due to van der Waals interactions. This agglomeration not only reduces the effective active interface of the material, but may also introduce micro defects in the matrix, thereby offsetting its inherent reinforcement advantages.^22^ Moreover, the dispersion stability of nanoparticles depends on the balance between attraction and electrostatic repulsion. The high alkaline environment formed by cement hydration contains a large amount of electrolytes such as calcium ions, which significantly weakens the electrostatic repulsion and makes van der Waals attraction dominant, ultimately leading to irreversible agglomeration of nanoparticles and difficulty in dispersion.^23^ Therefore, a deep understanding and effective regulation of the dispersion behavior of nanomaterials in cement systems is a key prerequisite for ensuring their full enhancement effect, and it is also a core issue facing research in this field.

Physical dispersion and chemical modification are two important methods for solving the agglomeration of nanomaterials in existing research. For example, using mechanical energy to break down aggregates through stirring or ultrasonic dispersion is a practical means of achieving initial dispersion.^24^ In addition, regulating the interactions between particles through chemical modification is also an effective method to solve agglomeration. For example, by introducing surfactants such as polycarboxylate superplasticizers into the system, their molecules can adsorb onto the surface of nanoparticles, forming a physical barrier between particles through steric hindrance effect to prevent them from approaching.^25^ Furthermore, by covalently functionalizing the surface of nanoparticles, their surface properties can be altered, enhancing their affinity for aqueous media and interfacial bonding with cement matrix, thereby further improving the stability and persistence of dispersion.^26^ It is worth noting that in recent years, research has explored a more promising strategy, which is to use a nanomaterial as a dispersion carrier to improve the dispersibility of other nanomaterials, providing new possibilities for synergistically enhancing cement-based composite materials. By leveraging the unique surface functional groups or morphological features of nanomaterials, they serve as “nano dispersed carriers” or “isolation layers” to insert and block other easily agglomerated nanoparticles.

It is noteworthy that scholars have found that GO can not only accelerate hydration reactions but also serve as a stabilizing dispersant.^27^ Studies have shown that the carboxyl, hydroxy, and epoxy groups on the surface of GO can interface with some nanomaterials, thereby significantly improving their dispersion in cementitious systems.^28^ He et al.^29^ showed that the dispersion of SiO_2_ and Al_2_O_3_ nanoparticles in the cementitious matrix could be effectively improved by 0.02% GO, and the compressive strength and flexural strength of the cementitious materials at 7 days of age were increased by 33.9% and 24.6%.

Therefore, the incorporation of multiple nanomaterials into cement mortar represents a cutting-edge strategy for achieving synergistic performance optimization. Since a single material often fails to comprehensively improve all properties, nanomaterials of different dimensions can exert complementary functional effects.^30^ For instance, when two-dimensional sheet-like GO is combined with zero-dimensional nano-SiO_2_, GO not only acts as a dispersion carrier but also works synergistically with nano-SiO_2_ to fill pores and accelerate hydration, leading to significantly enhanced strength and densification of the composite compared to using either material alone.^31^ This synergistic effect is also prominent in multidimensional hybrid systems constructed from GO and carbon nanotubes, where the cooperation between different materials enables the more effective formation of a reinforcing network.^32^ Consequently, the composite incorporation of nanomaterials provides a key pathway for designing high-performance cement-based materials with superior comprehensive properties.

Promoting the green and low-carbon transformation of the construction industry is a crucial topic for sustainable development. Cement production is one of the primary sources of carbon emissions, with its manufacturing process contributing to approximately 8% of global anthropogenic CO_2_ emissions.^33^ Therefore, enhancing the performance and efficiency of cement-based materials is essential for reducing the environmental footprint of construction. In particular, improving early-age mechanical properties can directly shorten the formwork turnover period and accelerate construction progress, thereby reducing on-site energy consumption and material waste, which offers clear sustainability benefits.^34^ Nanomaterials provide an effective pathway to achieve this.

Based on the above research background, this article takes cement mortar mixed with MLG as the basic system, further introduces GO to construct MLG/GO nanocomposite reinforcement system, and explores the synergistic enhancement effect of GO on the early performance of the system. This study systematically analyzed the influence mechanism of GO composite on the mechanical property evolution, hydration product generation, and microstructure compactness improvement of MLGs cement mortar from the perspectives of macroscopic mechanical properties (flow performance testing, flexural strength, and compressive strength), microstructure characterization (XRD, FT-IR, and SEM), and molecular dynamics (MD) analysis. By revealing the synergistic enhancement effect of nanomaterials mixed in cement matrix, we aim to provide reliable theoretical basis and experimental support for the early performance design and engineering application of high-performance green building materials. Conducting multi-scale research on the synergistic enhancement mechanism of MLGs/GO composite system not only helps deepen the understanding of the interfacial effects of nanomaterials in cement matrix, but also provides theoretical and experimental basis for the application of this system in modern engineering with strict requirements for early strength, such as prefabricated components, 3D printing, and rapid repair, and has clear engineering application value.

## Results

### Fluidity performance

**Figure 1:**
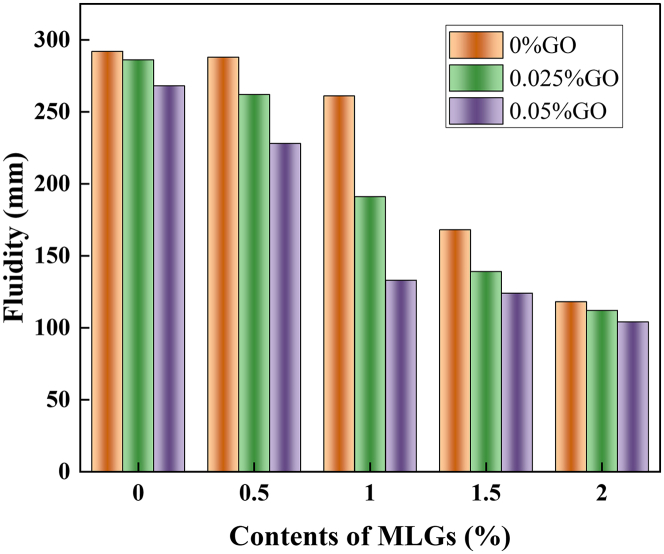
Fluidity of MLGs/GO hybrid mortar composites

The influence of GO introduction on the fluidity of hybrid mortar containing MLGs is illustrated in Figure 1. Since the specific surface area of MLGs and GO is larger than that of cement particles, both MLGs and GO can significantly reduce the fluidity of hybrid mortar composites. Taking Blank without GO and MLGs as the benchmark group, we observed that when 0.025% GO (L0-G025) or 0.5% MLGs (L050-G0) were added to the hybrid mortar composites, the fluidity decreased by 2.05% and 1.36%, respectively. Therefore, we believe that 0.025% GO and 0.5% MLGs have an equivalent effect on liquidity, and the liquidity loss caused by the two can be regarded as equivalent.

When the flow loss was maintained at 3 parts, compared with the cement mortar with MLGs alone, incorporating 0.025% GO markedly boosted the fluidity of hybrid mortar composites. More importantly, the enhancement of fluidity became increasingly pronounced as GO content rose from 0.025% to 0.05%. L100-G0 had a fluidity of 168 mm. When 0.025% GO was incorporated, the mortar’s fluidity increased by 13.69% relative to L100-G0. With the GO dosage going up from 0.025% to 0.05%, the fluidity was further boosted, reaching an increase of 35.71%. The results revealed that GO incorporation not only enabled effective MLGs dispersion and agglomeration inhibition but also maintained good dispersion stability without compromising mortar fluidity, ultimately enhancing fluidity.

When the flow loss was maintained at 4 parts, the addition of 0.025% GO markedly diminished the fluidity of hybrid mortar containing MLGs, and the fluidity of hybrid mortar composites decreased further as the GO dosages increased from 0.025% to 0.05%. The fluidity of hybrid mortar composites containing (L150-G0) alone MLGs was 146 mm, and the fluidity of hybrid mortar composites decreased by 4.79% after adding 0.025% GO. As the GO blend increased from 0.025% to 0.05%, liquidity decreased further, reaching 8.9%. It shows that under the condition of four flow losses, the dispersing ability of 0.025% GO diminishes, while its agglomeration tendency intensifies. As the GO dosage rises from 0.025% to 0.05%, its agglomeration is further intensified. This leads to more free water being adsorbed, increasing the water demand of hybrid mortar composites and thereby further reducing its fluidity.

### Flexural strength

**Figure 2:**
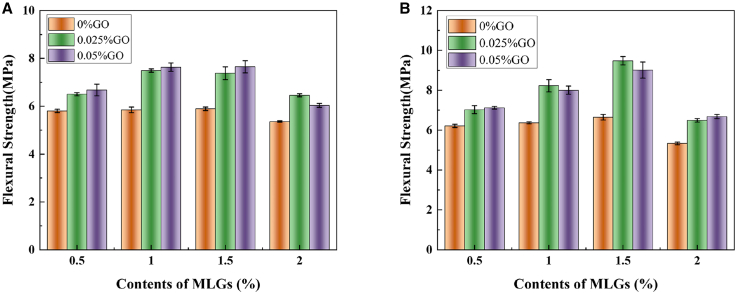
The effect of GO incorporation on the flexural strength of hybrid mortar composites containing MLGs (A) Hydration for 3 days and (B) hydration for 7 days.

The role of GO incorporation in affecting the flexural strength of hybrid mortar composites containing MLGs is depicted in Figure 2. The mortar’s flexural strength rose first and then declined as MLGs content increased. It shows that an appropriate amount of MLGs can effectively enhance the interfacial force of hydration products and improve the toughness of mortar. However, when the dosage is too high, MLGs are prone to agglomeration, resulting in the adsorption of excessive free water, inhibiting the hydration reaction, and forming pores between the hydration products, which ultimately leads to a decrease in flexural strength.

Upon adding 0.025% GO, the flexural strength of hybrid mortar composites containing MLGs was markedly enhanced, and the enhancement effect brought by the addition of one part of GO was much greater than that of adding one part of MLGs alone. Compared with the single mixed MLGs, 0.025% GO incorporation (L050-G025, L100-G025, L150-G025, and L200-G025) increased the flexural strength by 12.00%, 28.11%, 25.23%, and 20.58% on 3 days and 13.09%, 29.20%, 42.68% and 21.75% 7 days, respectively. It was shown that the introduction of 0.025% GO could effectively improve the dispersion of MLGs in the hybrid mortar composites, boost the compactness of the mortar, and enhance the interface force between MLGs and hydration products, thereby improving the flexural strength. With the increase of GO content from 0.025% to 0.05%, its effect on flexural strength tends to weaken, and even shows a downward trend in some cases. The flexural strength increased by 15.04%, 30.56%, 29.81%, and 12.62% on 3 days and 14.65%, 25.69%, 35.66% and 25.25% on 7 days, respectively. In the 1.5% and 2.0% MLGs-cement mortar (L150-G050, L200-G050) on 7 days, the flexural strength declined as GO content rose, implying that too much GO would lead to the agglomeration phenomenon in the cement matrix, which would lead to uneven distribution of local hydration products, increase porosity, and thus reduce the flexural strength.

### Compressive strength

**Figure 3:**
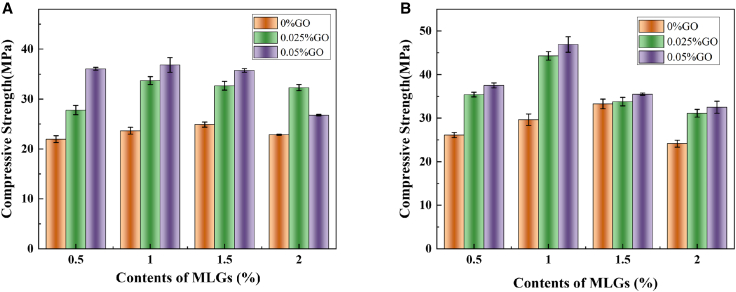
The effect of GO incorporation on the compressive strength of hybrid mortar composites containing MLGs (A) Hydration for 3 days and (B) hydration for 7 days.

The role of GO incorporation in affecting the compressive strength of hybrid mortar composites containing MLGs is depicted in Figure 3. Consistent with the flexural strength, the compressive strength of hybrid mortar composites containing MLGs at 3 and 7 days reached the maximum values of 24.89 MPa and 33.26 MPa as the MLGs dosage is 1.5%. The compressive strength went up initially before falling as MLGs content grew. This indicates that adding an appropriate amount of MLGs aids in boosting the mortar’s bearing capacity. Compared with the single MLGs (L050-G0, L100-G0, L150-G0, L200-G0), the incorporation of 0.025% GO increased the compressive strength by 26.48%, 42.49%, 31.27%, and 41.29% on 3 days, and increased by 35.67%, 49.50%, 1.61%, and 28.90% on 7 days. When the GO dosage raised from 0.025% to 0.05%, the compressive strength increased by 64.12%, 55.74%, 43.56%, 17.15% on 3 days, 43.72%, 58.28%, 6.73%, and 34.62% on 7 days, and reached the highest data of 36.83 MPa and 46.91 MPa at 1.0% MLGs, respectively. At the same time, we found that the compressive strength decreased in some groups at 3 and 7 days of age (such as L150-G050 and L200-G050), but it was generally higher than that of the single mixing group, indicating that GO could partially offset the negative effect of excessive incorporation of MLGs on strength, which may be related to the gradual filling of hydration products and the enhancement of interfacial effects.

In conclusion, the influence trend of GO on compressive strength is generally consistent with the change law of flexural strength, but there are still certain differences. The incorporation of 0.025% GO showed significant enhancement effects on both flexural strength and compressive strength, while the increase rate decreased when the dosage increased from 0.025% to 0.05% GO. It shows that GO incorporation can better exert the small-size performance of MLGs, formulate a networked structure within the cement matrix, and block the expansion of cracks, to improve the mechanical properties of mortar.

### Statistical analysis of mechanical properties

Statistical analysis (See Tables 1, 2, and 3) including ANOVA, correlation analysis, and partial correlation analysis systematically reveals the statistical relationships and mechanisms of MLGs and GO in the early-stage enhancement of cement mortar. The ANOVA results first indicate that the main effects of GO on both flexural and compressive strengths are highly significant, with F values ranging from 8.79 to 20.38 and corresponding *p* values generally below 0.02, suggesting that GO is a core variable driving the early performance improvement of the system. In contrast, the influence of MLGs shows clear selectivity, exhibiting a significant effect only on the 7-day flexural strength (F = 13.02, *p* = 0.005), while its impact on the 3-day compressive strength is not significant (*p* = 0.379). This implies that its enhancement mechanism may be more related to toughness improvement. Particularly important is that the interaction between MLGs and GO also reaches statistical significance (e.g., 7-day flexural strength F = 14.45, *p* = 0.003), preliminarily revealing a potential synergistic enhancement pathway between the two.

**Table 1:** ANOVA of experimental group

Variable	Flexural strengths		Compressive strengths	
	3 days	7 days	3 days	7 days
MLGs	F = 7.56435 (*p* = 0.01836)	F = 13.01754 (*p* = 0.0049)	F = 1.22719 (*p* = 0.37865)	F = 5.11928 (*p* = 0.04306)
GO	F = 20.37733 (*p* = 0.00211)	F = 16.59319 (*p* = 0.00359)	F = 12.8072 (*p* = 0.00684)	F = 8.79184 (*p* = 0.01647)
MLGs × GO	F = 12.68954 (*p* = 0.00383)	F = 14.4478 (*p* = 0.00271)	F = 5.85919 (*p* = 0.02629)	F = 6.58831 (*p* = 0.01997)

**Table 2:** Correlation analysis of experimental group

Variable 1	Variable 2		Pearson correlation coefficient (R)	*p* value
MLGs	Flexural strengths	3 days	−0.16605	0.67397
		7 days	−0.09636	0.54548
	Compressive strengths	3 days	−0.08834	0.80859
		7 days	−0.30743	0.63455
GO	Flexural strengths	3 days	0.60601	0.01625
		7 days	0.76578	0.06659
	Compressive strengths	3 days	0.78484	0.00145
		7 days	0.33103	0.02666

**Table 3:** Partial correlation analysis of experimental group

Variable 1	Variable 2		Control variable	Partial correlation coefficient (R)	*p* value
MLGs	Flexural strengths	3 days	GO	−0.22477	0.50639
		7 days		−0.11497	0.73641
	Compressive strengths	3 days		−0.15015	0.65946
		7 days		−0.39776	0.22571

To further clarify the direct relationships between variables, correlation analysis shows that GO exhibits a strong positive correlation with 3-day compressive strength (Pearson R = 0.785, *p* = 0.001) and is also significantly correlated with 3-day flexural strength (R = 0.606, *p* = 0.016). This supports the dominant role of GO in early strength development from the perspective of linear associations. In contrast, MLGs show no significant correlation with any strength indicators (all *p* > 0.50), suggesting that their individual contribution to strength development is limited. This finding aligns with the limited main effects of MLGs in the ANOVA, collectively pointing to the functional dominance of GO.

However, the above analysis does not yet account for potential interference between variables. Therefore, the study further introduces partial correlation analysis with GO as the control variable, aiming to isolate the contribution of GO and independently evaluate the net effect of MLGs. The results show that after controlling for GO, the partial correlation coefficients between MLGs and various strength indicators do not reach the significance threshold (e.g., 3-day flexural strength R = −0.225, *p* = 0.506), and the coefficients generally trend negatively. This key statistical evidence indicates that the enhancement effect of MLGs is not independent but highly dependent on the coexistence and synergy with GO. Without the dispersing effect and interfacial regulation of GO, the enhancement potential of MLGs in the system cannot be effectively realized, and it may even lead to slight negative effects due to agglomeration.

In summary, the series of statistical analyses progress step by step, collectively constructing a complete statistical interpretation of the MLGs/GO composite enhancement mechanism. GO, with its significant main effects and strong correlation with strength indicators, establishes its central role in early-stage enhancement. In contrast, the role of MLGs is subsidiary and conditional, with its effectiveness entirely dependent on interaction and synergy with GO. Therefore, in material design and optimization, GO should serve as the regulatory core. By fully leveraging its dispersing, nucleation, and interfacial strengthening functions, the enhancement potential of MLGs can be effectively activated, achieving systematic improvement in the early performance of composite materials.

### Crystal phase analysis

**Figure 4:**
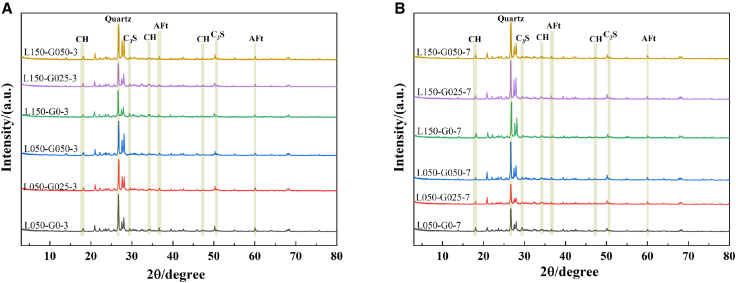
XRD spectra of hybrid mortar composites containing MLGs/GO (A) Hydration for 3 days and (B) hydration for 7 days.

The XRD spectra of GO on the early (3 and 7 days) hydration process of hybrid mortar composites containing MLGs are presented in Figure 4. As seen in the XRD patterns, no additional diffraction peaks are present in the crystal phase structure of different dosages of GO/MLGs cement mortar, indicating that the co-incorporation of GO and MLGs does not alter the structure and types of hydrated crystals in hybrid mortar composites. However, the strength of the unhydrated C_3_S crystal phase peak (29°, 50.37°) in the cement mortar with MLGs (L050-G0-3, L150-G0-3, L050-G0-7, and L150-G0-7) at 3 and 7 days of age was obvious, and the CH crystal phase peak (18°, 36°) of the secondary product produced by hydrated calcium silicate was weak, and the diffraction peak intensity decreased significantly with the extension of age from 3 days to 7 days. The results showed that the effect of single mixing of MLGs on stimulating the early hydration reaction of cementitious materials was limited.

When 0.025% GO was introduced, the intensity of C_3_S diffraction peak decreased significantly relative to the mortar test group’s mixed with MLGs, and the characteristic peaks of AFt and CH were significantly enhanced, and the degree of change gradually increased with the age from 3 days to 7 days, indicating that GO further provided a large number of nucleation sites for the hydration reaction on the basis of MLGs and accelerated the hydration reaction.

With the GO content increasing to 0.05%, the diffraction peaks of CH, AFt, and C3S saw a decrease in intensity, compared with L050-G025–3, L150-G025–3, L050-G025–7, L150-G025-7, CH, AFt, and C_3_S, indicating that with the further increase of GO content, the secondary hydration of hydration products was promoted in synergy with MLGs. Combined with the mechanical properties, it can be seen that the flexural and compressive strength of MLGs-cement mortar is further enhanced with the increase of GO content from 0.025% to 0.05%, indicating that the microstructure of the mortar is further optimized by triggering the secondary hydration reaction after the GO content is increased to 0.05%.

Ultimately, the incorporation of GO enhances the ability of MLGs as hydration templates and promotes the hydration of C_3_S to produce C-S-H. As the GO content rises from 0.025% to 0.05%, the synergistic effect between GO and MLGs is further boosted, which in turn improves the mortar’s mechanical performance.

### Infrared spectroscopy

**Figure 5:**
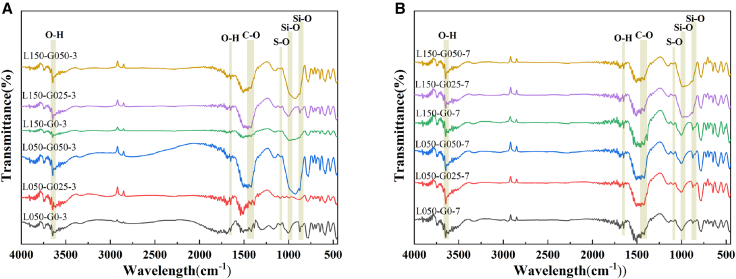
FT-IR of hybrid mortar composites containing MLGs/GO (A) Hydration for 3 days and (B) hydration for 7 days.

Figure 5 presents the FT-IR spectra of 0.5% and 1.5% MLGs-cement mortar remixing 0%, 0.025%, and 0.05% GO after 3 and 7 days of curing. It is apparent that L050-G0, L050-G025, L050-G050, L150-G0, L150-G0, L150-G025, and L150-G050 showed similar absorption peaks at both instars, and no additional absorption peaks were observed, signifying that incorporating GO exerted no impact on shifts in chemical bonds in hybrid mortar composites containing MLGs, consistent with XRD analysis results.

Specifically, the changes of each characteristic absorption peak were analyzed, and it was found that the incorporation of GO had a significant effect on the vibration absorption peaks of some bonds. Taking the O-H bond telescopic vibration (3,643 cm^−1^) of calcium hydroxide (CH) as an example, the absorption peak strength of the specimens mixed with 0.025% GO (L050-G025 and L150-G025) was stronger than that of the non-GO mortar specimens (L050-G0 and L150-G0) at three ages, indicating that the formation of CH increased with the incorporation of 0.025% GO. The O-H bond bending vibration peak (1,650 cm^−1^) of chemically bound water also showed significant enhancement, proving that after GO and MLGs were mixed, MLGs-cement mortar could store more chemically bound water, which was conducive to the long-term hydration reaction.

For the S-O bonded telescopic vibration absorption peak of calcium aluminate sulfate (AFt, 1,084 cm^−1^) and the Si-O bonded tetrahedral telescopic vibration absorption peak of hydrated calcium silicate gel (C-S-H gel, 1,000 cm^−1^), it was also observed that the addition of 0.025% GO brought stronger peak strength, indicating that the incorporation of GO significantly increased the formation rate of AFt and C-S-H gel in the early stage of hybrid mortar composites, and strengthened microstructure of hybrid mortar composites.

For both the S-O stretching vibration absorption peak of calcium aluminate sulfate (AFt, 1084 cm^−1^) and the Si-O tetrahedral stretching vibration absorption peak of calcium silicate hydrate gel (C-S-H gel, 1,000 cm^−1^), it was also noted that adding 0.025% GO resulted in stronger peak intensities. It can be attributed to the fact that the mutual enhancement effect of GO and MLGs effectively improved the compactness and reduced the intrusion of external CO_2_ and the residue of unhydrated particles.

When the GO content was increased to 0.05% and remixed with 1.5% MLGs, the intensity of the chemically bound water absorption peak (1,650 cm^−1^) was further increased, while the CH peak (3,643 cm^−1^) and the calcium carbonate peak (1,421 cm^−1^) weakened accordingly. This spectral change suggests a more advanced stage of hydration and microstructural evolution. The enhancement of the 1,650 cm^−1^ peak indicates a greater quantity of chemically bound water within the C-S-H gel, reflecting increased gel formation and improved microstructural densification. Concurrently, the attenuation of the CH peak does not necessarily imply a reduction in total CH content, but may result from the refined distribution and smaller crystal size of CH due to the templating effect of GO/MLGs, which can limit the detectability of characteristic O-H vibrations. Moreover, the suppressed carbonate peak suggests reduced carbonation, consistent with a denser matrix that hinders CO_2_ ingress. Thus, the collective FT-IR trends-enhanced bound water signal alongside attenuated CH and carbonate peaks—demonstrate that the higher GO dosage (0.05%) further promotes the formation of a dense, polymerized C-S-H network while refining the morphology and distribution of crystalline hydration products, thereby advancing the overall hydration degree and microstructural compactness.

Consequently, the co-incorporation of GO and MLGs markedly refines the microstructure of hybrid mortar composites, and promotes the development of early hydration efficiency and long-term characteristic of hybrid mortar composites by increasing the generation of CH, C-S-H gel, and AFt, and effectively storing chemically bound water, while reducing the formation of unhydrated particles and calcium carbonate, which promotes the development of early hydration efficiency and long-term characteristic of system, and provides a viable approach for the modification of hybrid mortar composites.

### Microscopic morphology analysis

**Figure 6:**
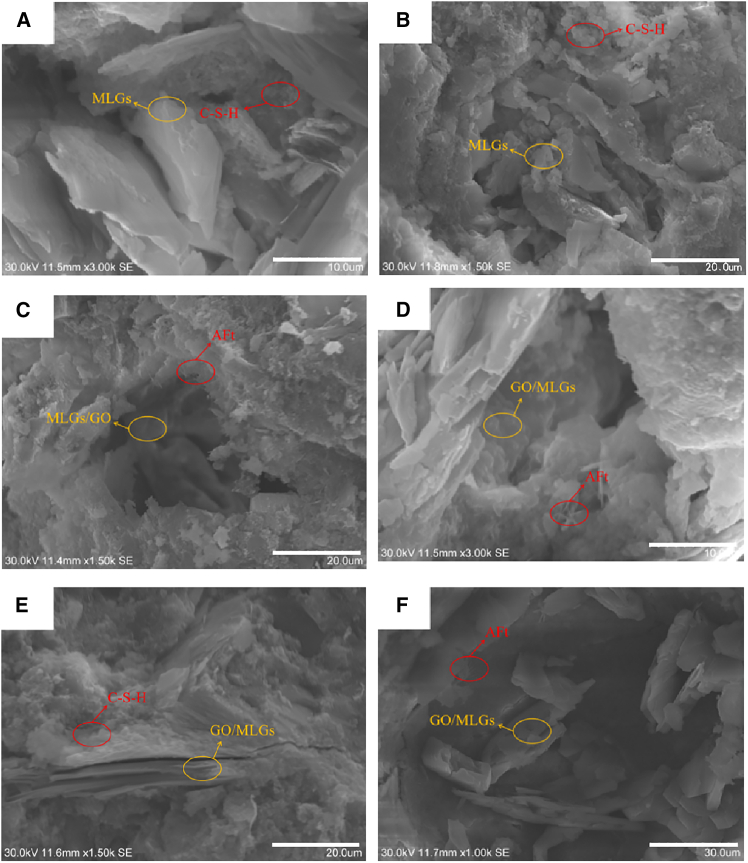
SEM images of hybrid mortar composites containing MLGs/GO at 3 and 7 days (A) L100-G0-3 days, (B) L100-G0-7 days, (C) L100-G025-3 days, (D) L100-G025-7 days, (E) L100-G050-3 days, and (F) L100-G050-7 days. The scale bars in (A) and (D) are 10 μm in length. The scale bars in (B, C, and D) are 20 μm in length.The scale bars in (F) is 30 μm in length.

SEM images of L100-G0, L100-G025, and L100-G050 at 3 and 7 days of age are displayed in Figure 6. In the microscopic diagram of the mortar test group with a single mix of 1.0% MLGs, the pores of the cement-based material in the 3-day age Figure 6A are filled by MLGs, and numerous flocculent C-S-H are attached to the MLGs. As the age period increased from 3 to 7 days, it can be observed from Figure 6B that the hydration products became more compact, the MLGs interspersed with each other, and the compactness of cement-based materials was significantly improved, but there were still microcracks interlaced in them.

With the addition of 0.025% GO to 1.0% MLGs, compared with the microscopic plot of mortar with 1.0% MLGs, the distribution of nanomaterials in Figure 6C became significantly more uniform, and the secondary product of hydrated calcium silicate also gradually increased, which verified that the incorporation of GO could help MLGs overcome van der Waals forces, reduce agglomeration, and accelerate the hydration process of cement. As the age period increased from 3 to 7 days, it can be observed from Figure 6D that the links between hydration products are tighter, and the microstructure is further improved compared with 3 days.

In Figure 6E of cement mortar mixed with 0.05% GO, we observed that some MLGs and GOs were stacked together, leading to microcracks in the microstructure of hybrid mortar composites, signifying that agglomeration triggered by excessive nanomaterials exerted an adverse effect on hybrid mortar composites. As the age period rose from 3 to 7 days (See Figure 6F), the negative effects of aggregation were alleviated, and the microstructure compactness was improved.

To sum up, the incorporation of MLGs and GO has a significant impact on the microstructure and hydration process of cement mortar. When 1.0% MLGs were added alone, it could effectively fill the pores and provide nucleation sites for hydration products, and although the density increased with the increase of age, there were still more microcracks. After re-mixing 0.025% GO and 1.0% MLGs, GO enhanced the interface with MLGs through oxygen-containing functional groups, improved the dispersion of nanomaterials, accelerated the cement hydration reaction, and significantly optimized the microstructure uniformity and density of early age. However, when the GO mixing increased to 0.05%, excessive nanomaterials triggered agglomeration, which brought negative effects. Although some of the negative effects were alleviated by hydration product filling with age, the overall structure was still lower than that of the 0.025% GO dosage group. This indicates that reasonable regulation of the content and ratio of MLGs and GO in cement mortar is crucial for optimizing the microstructure and improving the material properties.

The typical morphologies of different hydration products can be clearly identified from the SEM images: flocculent or reticulated structures correspond to C-S-H gel, needle-like or rod-shaped crystals are AFt, and plate-like or layered crystals correspond to CH. The introduction of GO significantly modulates the morphology and spatial distribution of these products. In the L100-G025 group, C-S-H exhibits a denser and more continuous network structure, AFt crystals show more uniform size and better dispersion, and CH crystals are reduced in size and more evenly embedded within the C-S-H network. These morphological features directly correlate with the enhancement of characteristic peaks for CH and AFt in FT-IR and XRD, as well as the accelerated consumption of C3S, indicating that the MLGs/GO composite system not only promotes the hydration process but also effectively regulates the microscopic morphology of the products.

The synergistic effect of MLGs and GO is manifested in several aspects. The oxygen-containing functional groups on the GO surface enhance interfacial bonding with MLGs, leading to more uniform dispersion of MLGs, which act as physical scaffolds to guide the directional growth of C-S-H gel and bridge micro-pores. Simultaneously, the nucleation sites provided by GO, combined with the templating effect of MLGs, jointly inhibit the excessive growth and localized aggregation of CH and AFt, thereby optimizing the uniformity and compactness of the microstructure. However, when the GO dosage is excessive (e.g., L100-G050), the stacking of nano-sheets intensifies, hindering the complete encapsulation of hydration products around MLGs and resulting in localized interfacial weak zones and microcracks. This observation aligns with the trend of decreased macroscopic mechanical performance and increased porosity.

In summary, through morphological regulation and chemical synergy, MLGs/GO not only accelerate early-age hydration but, more importantly, improve the spatial distribution and interfacial bonding of hydration products, thereby providing a microstructural foundation for achieving significant enhancement in the early mechanical properties of mortar.

### Strain bonding energy

**Figure 7:**
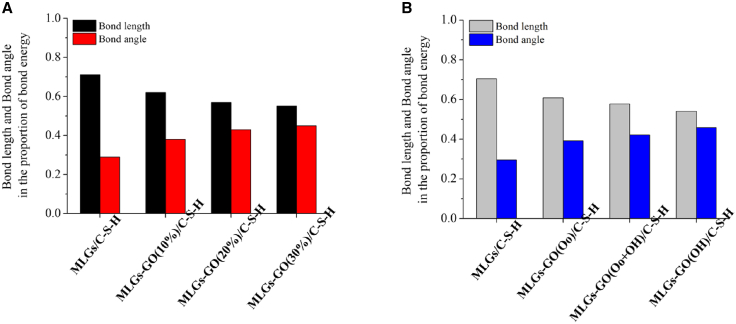
Bond length and bond angle in the proportion of bond energy (A) MLGs/C-S-H with different contents of GO functional group and (B) MLGs/C-S-H with different kinds of GO functional group.

Figure 7 shows bond length and bond angle with respect to the share of bond energy in MLGs/C-S-H with different cases of GO nanostructures. It is shown from Figure 7A that the pristine MLGs/C-S-H nanostructures exhibited a highest bond length value of 0.711 in the proportion of bond energy, while it showed a lowest bond angle value of 0.289 in the proportion of bond energy. There is a larger difference value (*β* = *ϑ*-ϒ, 0.422) between *ϑ* and ϒ value in pristine MLGs/C-S-H nanostructures. From the perspective of the principle of minimum energy, external stress may force the bond length to remain relatively stable (such as the rigid limitation of covalent bonds), but the bond angle is forced to deviate from the ideal value (such as bending or torsion), and the negative influence of bond angle on bond energy (tension energy) will increase significantly, resulting in its “negative contribution ratio” in bond energy being much greater than the bond length; If the contribution ratio between them is unbalanced, it may indicate that there is forced distortion in the structure.

The above phenomenon will be changed when GO layer is added. Incorporation of GO layer decreased *β* values by 43.13%, 66.82%, and 76.30% for the corresponding MLGs-GO/C-S-H hybrid systems with 10%, 20% and 30% GO. Clearly, the *β* values of MLGs/C-S-H hybrid systems decrease with increase of GO contents, and the *β* values dependent on the GO contents.

Evidently, the variation in bond angles (ϒ) dominates the bond energy percentage within MLGs-GO/C-S-H hybrid systems, while ϒ contributions remain relatively minor in MLGs/C-S-H nanostructures. This shows that MLGs-GO/C-S-H hybrid systems have more uniform bond energy distribution and smaller internal stress, compared with MLGs/C-S-H nanostructures.

Next, we pay attention to the effect of different kinds of GO functional groups on *ϑ* and ϒ of MLGs/C-S-H nanostructures, as displayed in Figure 7B. For MLGs/C-S-H incorporating hydroxyl-functionalized GO (MLGs-GO(OH)/C-S-H), the *ϑ* value is lower than that of MLGs/C-S-H nanostructures, while its ϒ value is highest. The *β* value of MLGs/C-S-H nanostructures decreases with adding the functional group of GO. In addition, in terms of *β*, the performance of the system containing only hydroxyl groups is better than that containing both hydroxyl groups and epoxy groups, which is better than that containing only epoxy groups. The influence of hydroxyl groups in GO on the *β* of MLGs/C-S-H nanostructures is more significant than that of epoxy groups, because the hydroxyl groups have stronger polarity, richer bonding modes, smaller space resistance and better GO dispersion adjustment. Of course, adding hydroxyl group or epoxy group has a better effect on *β* value than not adding GO layer. With the addition of GO layer, the weak vdW between MLGs and C-S-H matrix becomes a strong non-covalent interaction.

Specifically, in the MLGs/C-S-H system without GO incorporation, the interface is primarily governed by weak van der Waals interactions, resulting in a highly concentrated and non-uniform bond-energy distribution. In real cementitious microstructures, such an interfacial state manifests as a stress-concentration zone and a preferential site for microcrack initiation. Under external loading, interfacial sliding or debonding is therefore prone to occur, which limits the effective load-bearing capacity and reinforcing efficiency of MLGs.

After the introduction of GO, the oxygen-containing functional groups on its surface form strong non-covalent interactions with the C-S-H matrix, leading to a more uniform redistribution of interfacial bond energy and a pronounced reduction in local stress concentration. From a microstructural perspective, this indicates the formation of a more continuous and mechanically stable transition interface between MLGs/GO and the hydration products, enabling the nanoreinforcement to participate in cooperative load transfer rather than acting merely as an embedded inclusion. This process corresponds, in real cement microstructures, to a transfer of stress from localized high-energy bonds to more stable interfacial bonding networks. Physically, such redistribution delays the rupture of critical bonds, thereby enhancing deformation compatibility and energy dissipation capacity. From an engineering standpoint, this atomistic energy-regulation mechanism can be interpreted as microscale stress redistribution and damage mitigation within the cement matrix, which provides a mechanistic explanation for the experimentally observed improvements in strength, ductility, and crack-propagation resistance.

## Discussion

This study systematically revealed the synergistic enhancement mechanism and optimal ratio of MLGs/GO nanocomposites on the early performance of cement mortar by constructing a multi-scale research method.

The experimental results indicate that the combined incorporation of MLGs and GO significantly synergistically enhances early strength. Among them, a GO content of 0.025% is identified as a critical threshold. At this dosage, GO effectively optimizes the dispersion of MLGs and maximizes the interfacial reinforcement effect. The optimal mix ratio (1.0% MLGs +0.025% GO) achieves a 7-day compressive strength of 46.91 MPa for the mortar, representing an over 58% improvement compared to the single MLGs group. When the GO content increases to 0.05%, the reinforcement efficiency declines due to intensified nanoparticle agglomeration.

The synergistic enhancement mechanism revealed in this study follows a clear multiscale pathway. The primary role of GO introduction is as an efficient nanodispersant, which effectively suppresses MLG agglomeration through steric hindrance and electrostatic repulsion effects, thereby achieving uniform distribution of nano-enhanced phases within the cement matrix. This optimized physical distribution lays the foundation for subsequent processes. The uniformly dispersed MLGs and GO jointly provide abundant heterogeneous nucleation sites, a conclusion supported by XRD and FT-IR analyses, which synergistically accelerate the early hydration of cement minerals, ultimately guiding the formation of denser and more uniform microstructures, as confirmed by SEM observations. Crucially, MD simulations elucidate the essence of interfacial reinforcement at the atomic scale: after GO sheet insertion, the oxygen-containing functional groups on its surface form strong non-covalent interactions with the C-S-H matrix, replacing the original weak van der Waals forces. This fundamental interfacial reconstruction optimizes the local bonding environment and stress distribution, significantly enhancing interfacial stress transfer efficiency. This quantitative mechanistic explanation clarifies why 0.025% GO serves as the optimal dosage: this proportion precisely strikes the ideal balance between forming a complete and robust interfacial layer and preventing GO sheets from self-aggregating due to excessive loading.

This study still has certain limitations. Firstly, the dispersion state and interfacial chemical evolution process of nanocomposites in cement slurry still lack advanced characterization support *in situ* and quantitatively, such as Raman surface scanning and *in situ* transmission electron microscopy, making it difficult to accurately analyze the deep correlation between their “dispersion performance.” Secondly, the conclusions of this study are mainly based on early performance data under standard curing conditions. The impact of this composite system on the long-term durability of cement-based materials, such as shrinkage, impermeability, and corrosion resistance, still needs to be systematically investigated. In addition, the current experiment is only conducted within a single cement system, and the applicability of its conclusions in composite systems containing mineral admixtures or complex environments such as temperature changes and wet dry cycles needs further verification. In response to the above limitations, future research should be promoted in the following areas. By introducing advanced *in situ* characterization methods, the structural evolution and spatial distribution behavior of nanomaterials during cement hydration process can be quantitatively tracked; simultaneously evaluate the impact of the composite system on the long-term performance and durability of mortar and concrete; and further expand to different cementitious systems and environmental conditions closer to actual service, in order to comprehensively examine its technical feasibility and engineering applicability.

### Limitations of the study

First, while the MD analysis provides valuable comparative insight into interfacial stress redistribution, the adopted indicators are not intended to represent absolute macroscopic failure criteria, and effects such as long-range diffusion, chemical reactions beyond non-covalent interactions, and time-dependent damage evolution were not explicitly included in the modeling.

Although the MD simulations and the macroscopic experiments exhibit consistent overall trends, the two approaches are not parameterized in a one-to-one manner. In MD, the model is typically constructed by prescribing the spatial proportion, geometry, and relative distribution of phases (i.e., a space-filling or interfacial-area-based representation of the nano-phase), whereas the experiments control the nano-additives by their dosage (mass fraction or volume fraction added to the cementitious system). The mapping from “dosage” to “effective spatial proportion/network morphology” is strongly influenced by dispersion quality, agglomeration, orientation, pore structure, and hydration-product growth. Consequently, differences may remain in quantitative magnitudes, threshold locations, and the fine details around the optimum region, even when the qualitative tendencies agree.

## Resource availability

### Lead contact

Further information and requests for resources and information should be directed to and will be fulfilled by the lead contact, Lei Fan, (121099@zust.edu.cn).

### Materials availability

This study did not generate new unique reagents.

### Data and code availability

• Raw data reported in this article will be shared by the lead contact upon request.
• This article does not report the original code.
• Any additional information required to reanalyze the data reported in this article is available from the lead contact upon request.

## Acknowledgments

This research was supported by the Funds of the Natural Science Foundation of Hangzhou under grant no. 2024SZRYBE050002, 10.13039/501100004731Natural Science Foundation of Zhejiang Province (LQ23E080003), Scientific and Technological Projects Entrusted by Enterprises and Institutions (HKJ20250027, HKJ20240094, and HKJ20250121).

## Author contributions

Conceptualization, L.F., G.L., and F.S.; methodology, F.L., L.U., X.J., and W.Z.; software, L.F. and H.W.; formal analysis, L.F. and J.Z.; resources, L.F.; data curation, C.W. and J.Z.; writing – original draft, L.F., C.W., and J.Z.; writing – review and editing, L.F.; visualization, Y.T. and Q.W.; supervision, M.L.; project administration, L.F.; funding acquisition, L.F.

## Declaration of interests

The authors declare no competing interests.

## STAR★Methods

### Key resources table

**Table:** 

REAGENT or RESOURCE	SOURCE	IDENTIFIER
**Software and algorithms**		

LAMMPS	Open source	www.lammps.org
Origin version 2019b	Smith et al.^35^	https://www.originlab.com
Ovito	Stukowski et al.^36^	https://www.ovito.org/

### Method details

#### Materials preparation and methods

In this test, Conch brand P.O 42.5 ordinary Portland cement was selected, and its specific chemical composition is listed in Table S1. The river sand used had a particle size distribution ranging from 0.01 to 4 mm, with a fineness modulus of 2.7. The water-reducing agent was FK-A type polycarboxylate high-performance water-reducing powder, which achieved a water reduction efficiency of up to 26%. Ordinary tap water was used as the mixing water. The graphene oxide used in the test was SH-GO-1280 type, synthesized via the modified Hummers method, with a thickness of approximately 1 nm, an oxygen content exceeding 54%, a purity of 99.9%, and an exfoliation rate of 99%. Multilayer graphene of the HR15-16 type was employed, with no less than 3 layers, a density ranging from 0.5 to 0.8 g/cm^3^, and a moisture content of 0.02%.

The GO dispersion was prepared using the ultrasonic dispersion method, with polycarboxylate superplasticizer incorporated to assist in the dispersion process. First, the superplasticizer powder and water were mixed in a specific ratio and stirred at 500 r/min for 15 minutes at 25°C until clear. Subsequently, GO was added, and the stirring speed was increased to 800 r/min for an additional 30 minutes. The mixture was then subjected to ultrasonic treatment (300 W, 40 kHz) in an intermittent mode for a total of 60 minutes, with the temperature controlled to ≤40°C. After washing, filtration, and freeze-drying, modified GO was obtained, and its morphological characteristics are shown in Figure S1(a-b). The modified GO and MLGs were then dissolved in water according to the specified proportions (see Figure S2) and subjected to the same dispersion process to form a homogeneous suspension. The mix design of this study is based on the aforementioned theoretical considerations, aiming to systematically evaluate the synergistic enhancement mechanism of MLGs and GO. The MLGs dosage was selected as 0.5%, 1.0%, 1.5%, and 2.0% to cover the whole process from effective enhancement to agglomeration-induced deterioration. The GO dosage was set at 0.025% and 0.05%, representing the critical range between dispersion promotion and agglomeration risk. This design not only allows verification of the peak dosage effect of MLGs, but also clarifies the “nano-carrier” function of GO at low dosages and its self-agglomeration tendency at higher dosages.

High-speed shearing was further applied to prevent agglomeration. In the mortar preparation stage, cement, water, standard sand, and the nano-suspension were mixed. Low-speed stirring was initially used to form a paste, followed by the addition of river sand (binder-to-sand ratio of 1:2). The mixture was stirred in two stages, first at low speed and then at high speed, until uniform. The prepared mixture was poured in two layers into 40×40×160 mm^3^ molds, compacted, leveled, and allowed to set. After demolding, the specimens were cured under conditions of 20±1°C and ≥95% humidity until the designated testing ages.

#### Testing and characterization of materials

Raman imaging spectrometer (THERMO NICOLET IS50) and X-ray diffractometer (Rigaku Ultimate IV) were used to analyze the hydration production of hybrid mortar nanocomposites; scanning electron microscope (SU1510) was employed to characterize the morphology of the hybrid mortar nanocomposites.

The flexural strength and compressive strength of the hybrid mortar nanocomposites were tested in accordance with the requirements of (GB/T 17671-2021, ISO Method). The loading rate of the universal testing machine was set to 0.5 kN/s. The fluidity performance of hybrid mortar nanocomposites was characterized according to GB/T 2419.

#### Computational model and method

High-performance atomic-scale particle interaction simulations were carried out using LAMMPS, a specialized modeling tool for molecular dynamics studies,^6^^,^^37^ with subsequent data visualization executed via the analytical mapping functionalities of the Origin platform.^35^ During the preparatory stage of molecular modeling, the three-dimensional structures of calcium silicate hydrate (C-S-H), MLGs, and GO were digitally reconstructed utilizing the atomic-level design module within Materials Studio.^38^^,^^39^

The main bonding phase C-S-H in cement materials has an initial configuration derived from the 11 Å Tobermorite lattice. This naturally occurring calcium silicate hydrate mineral is acknowledged as a structural analogue, effectively resembling the hydration products of cementitious materials.^40^^,^^41^ Through the incorporation of extra-calcium ions, the final arrangement of silicate polymerization matches the distribution observed in NMR spectroscopy.^42^^,^^43^

To compare the mechanical property differences between the reference group (MLGs/C-S-H) and the control group (MLGs-GO/C-S-H), as well as their reinforcing effects on the C-S-H matrix, two layers of graphene oxide were inserted into the MLGs/C-S-H system, as displayed in Figure S3. Subsequently, the impact of MLGs on the interlayer properties of nanocomposites was investigated under varying conditions: different GO contents (0%, 10%, 20%, and 30%), and different GO types (hydroxyl-only, epoxy-only, and hydroxyl+epoxy).

Prior research has confirmed that the Reactive force field is highly adept at depicting elements (calcium, silicon, oxygen, carbon, hydrogen), with particular relevance to systems: GNS, C-S-H gel, Tobermorite structure, and GO/C-S-H layered configuration.^44^^,^^45^

In the present study, we select AIREBO potential to characterize the covalent and vdW interactions within the molecular structures of CNTs and graphene.^46^^,^^47^ Additionally, the long-range interactions are described by the LJ potential. Additional in-depth information about the LJ potential is available in references.^48^^,^^49^ In the simulation work, the NPT ensemble is adopted. Furthermore, boundary conditions are implemented throughout the entire simulation process. To be specific, the temperature is fixed at 293 K and pressure held at 1 atm. Each step has a length of 1 fs, and the overall simulation totals 10 ns in duration.

#### Strain bonding energy

The interlayer stress transfer efficiency shows a strong correlation with the change of bond geometry (including bond angles and lengths) caused by GO within MLGs/GO layered nanostructures. Based on the theory of distorted stresses and bonding energies, these structural alterations-including variations in bond angles (*θ*_*angle*_), bond lengths (*r*_*bond*_), and bond energies can be quantitatively characterized through Equations 1-2:

(Equation 1) $$r_{bond}=q\sum_{m=1}^{3}\int_{r_{0}}^{r_{x}}\frac{1}{2}f_{MLGs}\;\sin\;\theta_{MLGs}dr_{MLGs}+n\sum_{m=1}^{3}\int_{r_{0}}^{r_{x}}\frac{1}{2}f_{GO}\;\sin\;\theta_{GO}dr_{GO}$$

(Equation 2) $$\theta_{angle}=i\sum_{j=1}^{3}\int_{\theta_{0}}^{\theta_{x}}M_{MLGs}\theta_{MLGs}d\theta_{MLGs}+k\sum_{j=1}^{3}\int_{k_{0}}^{k_{x}}M_{GO}\theta_{GO}d\theta_{GO}$$

Where *M*_*MLGs*_ and *M*_*GO*_ are the bending moment arising from covalent bond angle deformation of MLGs and GO layers. *f*_*MLGs*_ and *f*_*GO*_ represent the forces acting on the covalent bonds of MLGs and GO layers; *r*_0_ and *r*_*x*_ denote the bond lengths of atoms before and after force application, respectively. *θ*_0_ and *θ*_*x*_ correspond to the bond angles of atoms before and after atomic force application, respectively.

Through the application of Equations 1-2, we quantitatively evaluate the variations in bonding angles and lengths correlated with the bond energy redistribution.

(Equation 3) $$\vartheta =\frac{r_{bond}}{r_{bond}+\theta_{angle}}$$

(Equation 4) $$ϒ=\frac{\theta_{angle}}{r_{bond}+\theta_{angle}}$$

(Equation 5) β=ϑ−ϒ

Where *ϑ* and ϒ are the variations percentage in bond length and bond angle of the MLGs/GO layer with respect to the proportion of bond energies, respectively.

It should be noted that these equations do not aim to provide absolute fracture or failure criteria at the macroscopic scale. Instead, they serve as comparative indicators to evaluate trends in interfacial stress redistribution and bonding uniformity under different nanoscale interfacial architectures. Effects such as long-range diffusion, chemical reactions beyond non-covalent interactions, and time-dependent damage evolution are not explicitly considered and represent limitations of the present modeling framework.

### Quantification and statistical analysis

Mechanical performance (fluidity, flexural strength, and compressive strength) was quantified following the corresponding Chinese/ISO standards described in the Methods section, and the reported values represent aggregated results at each curing age (3 days and 7 days).

For inferential statistics, a factorial analysis of variance (ANOVA) was used to assess the main effects of MLGs dosage, GO dosage, and their interaction on flexural and compressive strengths at different ages; F statistics and associated P values were reported to determine statistical significance.

To further evaluate linear associations between material factors and strength outcomes, Pearson correlation coefficients (R) and P values were calculated. In addition, partial correlation analysis was performed by controlling GO as a covariate to isolate the net contribution of MLGs when potential confounding by GO exists. Unless otherwise specified, statistical significance was evaluated based on P values, consistent with the reporting in the manuscript tables and text.

For molecular dynamics (MD) simulations, trajectories were generated using LAMMPS under the specified ensemble and simulation settings, and the derived structural/mechanical descriptors were quantitatively extracted from the atomic configurations and visualized/processed using Origin as described.
