# Supplementary material for: Non-covalent interface engineering of multi-layer graphene cement composites using graphene oxide
Source: iScience. 2026 Feb 17;29(3):115065. doi: 10.1016/j.isci.2026.115065 (PMC12969085; doi:10.1016/j.isci.2026.115065)
Supplement: Document S1. Figures S1–S3 and Table S1 [file mmc1.pdf]

iScience, Volume *29*

Supplemental information

Non-covalent interface engineering of multi-layer graphene cement composites using graphene oxide

Lei Fan, Chengtao Wu, Jinhao Zheng, Xiaohan Ji, Wang Zhang, Yunyun Tong, Qiannan Wang, Fangyuan Song, Hongwei Wang, Feng Li, Lucas Uzimaya, Guangyan Liu, and Mengya Li

Supplementary materials

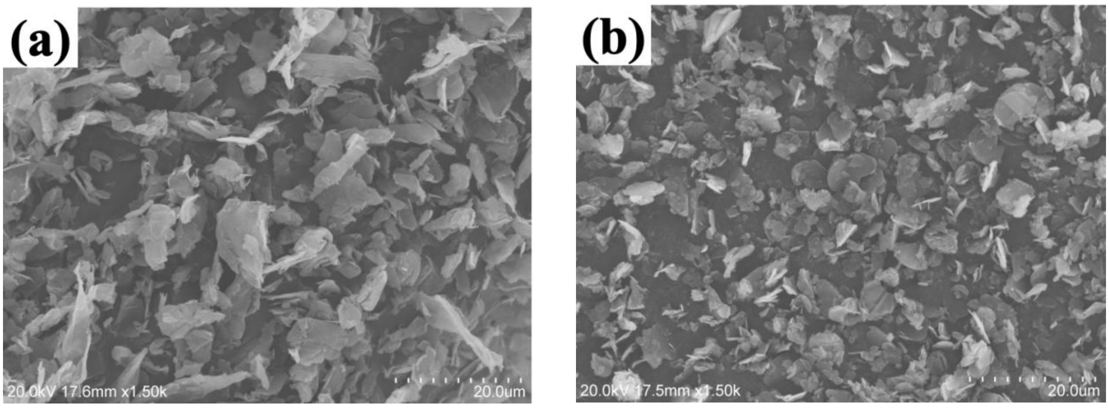

Figure S1. Microstructure of materials. (a) Microstructure of MLGs and (b) Microstructure of GO.

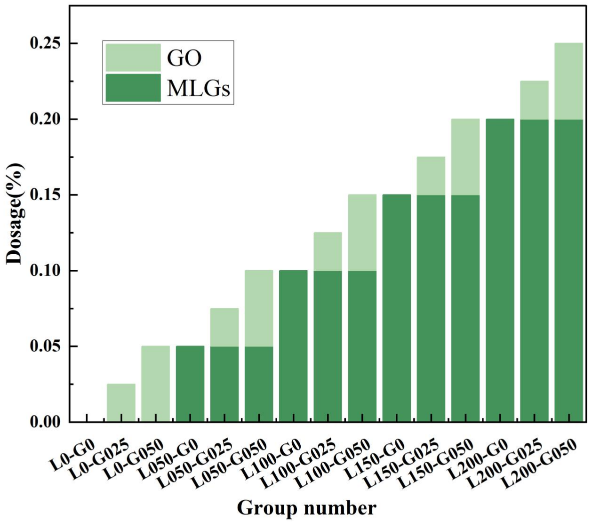

Figure S2. The mix proportion design of MLGs/GO experimental group.

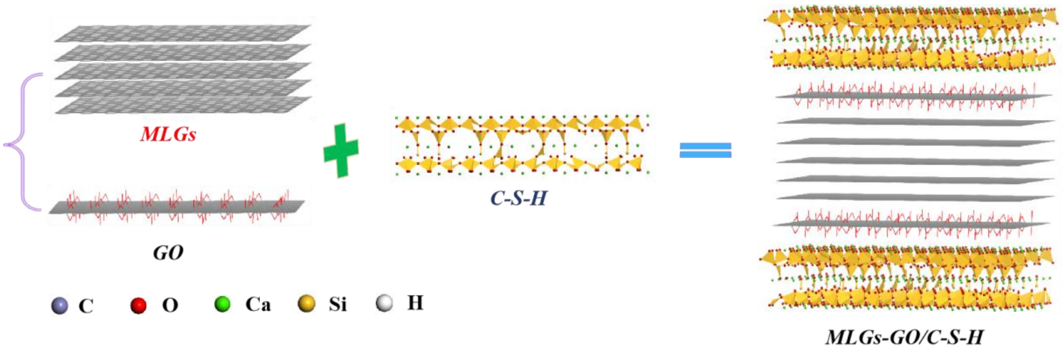

Figure S3. The pictorial representation of the development of MLGs-GO/C-S-H atomistic structures.

Table S1.Cement chemical composition list.

molecular formula	CaO	SiO_2	Al_2O_3	SO_3	Fe_2O_3	K_2O	MgO
content	54.36%	22.31%	9.76%	3.16%	3.13%	1.03%	1.01%
